# Promoting Cross-Racial and Ethnic Friendships in Schools: Roles of School Diversity and Interracial Climate and Intersections with Immigrant Status

**DOI:** 10.1007/s10964-025-02182-z

**Published:** 2025-04-03

**Authors:** Mei-ki Chan, Aprile D. Benner

**Affiliations:** 1 Department of Psychology, Utah State University, Logan, UT USA; 2 Department of Human Development and Family Sciences, University of Texas at Austin, Austin, TX USA

**Keywords:** Friendship, School diversity, Interracial climate, Immigrant students, Cross-racial/ethnic interactions

## Abstract

Cross-racial/ethnic friendships are associated with positive outcomes related to social cohesion; however, attention to the specific school contextual factors that promote these friendships during adolescence and how such factors vary by adolescents’ social positions is lacking. This study examined how school diversity and interracial climate were related to students’ friendship diversity and whether these associations differed by immigrant status. The participants were from a diverse sample of 591 U.S. 9th graders who were approximately 14- to 15-year-old across 29 schools (10% Asian American, 4% Black, 34% Latino/a/x, 40% White, and 12% other or multiple races/ethnicities; 53% female). The results indicated that higher school racial/ethnic diversity was linked to greater friendship diversity. However, this relation diminished as school diversity increased and was less pronounced among adolescents from immigrant families. Youth from immigrant families who perceived a more positive interracial climate among peers reported having more diverse friendships compared to their counterparts from immigrant families in the same schools. The findings highlight the facilitating roles of school diversity and peer interracial climate in positive interracial interactions and the varying influences of adolescents’ immigrant status.

## Introduction

Cross-racial/ethnic friendships represent a form of positive intergroup interactions that have been shown to reduce prejudice (Chavez et al., 2021) and support students’ positive school adjustment, particularly for immigrant students (Schachner et al., 2018). These interactions are especially critical in today’s school contexts, as U.S. public schools serve an increasingly diverse student population, with over half of students identifying as racially and ethnically minoritized (National Center for Education Statistics, 2024) and a growing number coming from immigrant backgrounds (Center for Immigration Studies, 2023). Promoting cross-racial/ethnic friendships, however, requires intentional efforts because adolescents, particularly those from immigrant families, show a persistent preference for making friends with same-racial/ethnic peers (Joyner & Kao, 2000; Titzmann & Silbereisen, 2009). Despite the importance of cross-racial/ethnic friendships, several research gaps remain. Evidence regarding the impact of school diversity on cross-racial/ethnic friendships is conflicting, with studies documenting both positive and negative effects (Fischer, 2008; Kogachi & Graham, 2021) and largely overlooking the potential for non-linear relations. Moreover, little is known regarding how malleable contextual factors, such as perceived school climate, facilitate cross-racial/ethnic friendships and how such influences may differ based on adolescents’ social positions, such as immigrant status (Titzmann, 2014). To address these gaps, the current study has two purposes. First, this study examines whether and how school diversity relates to friendship diversity and whether the association between school diversity and cross-racial/ethnic friendships varies across adolescents from immigrant versus non-immigrant families. The second purpose is to evaluate the role of school interracial climate in cross-racial/ethnic friendships and examine if this relation likewise depends on adolescents’ immigrant status. These associations were analyzed within a multilevel framework to distinguish the effects on cross-racial/ethnic friendships at the school and student levels using a sample of U.S adolescents.

### Cross-Racial/Ethnic Friendships

Cross-racial/ethnic friendships yield significant benefits for adolescents and society. For adolescents, being friends with individuals from different racial and ethnic backgrounds has been linked to reduced relational victimization (Kawabata & Crick, 2011) and enhanced perceived school safety (Chen & Graham, 2017). Among students with culturally marginalized identities, cross-racial/ethnic friendships have been associated with more positive socioemotional development (Kelleghan et al., 2019; Liu et al., 2020) and less perceived vulnerability in schools (Graham et al., 2014). Moreover, the positive effects of cross-racial/ethnic friendships extend beyond individual well-being. Documented linkages of cross-racial/ethnic friendships with more favorable intergroup attitudes (Chen & Graham, 2017; Kelleghan et al., 2019), greater inclusivity (Kawabata & Crick, 2008), and reduced prejudice (Ahmad et al., 2018) highlight the crucial role that cross-racial/ethnic friendships play in fostering social cohesion.

Despite the extensive benefits of cross-racial/ethnic friendships, adolescents have been consistently observed to gravitate toward making friends who share their same-race/ethnicity (Kogachi & Graham, 2021), meaning that intentional strategies may be necessary to encourage and thus reap the benefits of cross-racial/ethnic friendships. Several theoretical rationales, including person-context fit theory and similarity, have indicated that individuals tend to form friendships with others sharing more similarities with them (Magnusson & Stattin, 1996; Urberg et al., 1998). Given the advantages of cross-racial/ethnic friendships for adolescents and their potential for enhancing long-term social harmony and inclusion, further research into strategies promoting cross-racial/ethnic friendships in schools is needed.

### The Role of School Diversity

Several theories point to the significant role of school racial and ethnic composition in cross-racial/ethnic friendships formation but differ in the expected impacts. One line of theory and research suggests that diverse contexts promote cross-racial/ethnic friendships. Social contact theory asserts that a diverse context provides opportunities for intergroup contact, with positive contact more likely to occur when there is power balance across groups (Pettigrew et al., 2011). One critical factor shaping perceived power dynamics across groups is numerical representation (i.e., the proportion of a racial and ethnic group within a context); equal group distributions often equate to a more balanced power structure (Pettigrew et al., 2011). Therefore, schools with more equally distributed racial and ethnic groups (i.e., higher school diversity) likely maximize the opportunities for individuals to make friendships with peers from more racially and ethnically diverse backgrounds. Some studies have observed a positive relation between school diversity and cross-racial/ethnic friendships. For instance, higher school diversity was associated with increased friendship diversity among White and non-White university students (Fischer, 2008), and a recent study of U.S. high school students also observed that students attending more diverse schools developed and maintained higher cross-racial/ethnic friendships over time (Lorenzo et al., 2024). A study of non-immigrant Swedish youth similarly found that students in classrooms with more immigrant adolescents had a higher proportion of close friends from immigrant backgrounds than those in classrooms with a low proportion of immigrant adolescents (Bohman & Miklikowska, 2021).

In contrast, other theories and empirical studies suggest that diverse contexts can inhibit the development of cross-racial/ethnic friendships. Intergroup conflict and constrict theories propose that students are less likely to form cross-racial/ethnic friendships in schools with higher racial and ethnic diversity (Blalock, 1967; Putnam, 2007). Based on constrict theory, ethnic diversity would hamper social cohesion because people tend to “hunker down” in a diverse context due to less perceived trust (Putnam, 2007). Likewise, conflict theory proposes people perceive increased intergroup threats and favorable in-group bias in more diverse contexts (Blalock, 1967). Some prior studies across settings have corroborated these theoretical propositions. For example, recent research involving middle school students in the Netherlands and the U.S. observed that higher classroom and school diversity were associated with fewer cross-racial/ethnic friendships (Kogachi & Graham, 2021; Munniksma et al., 2017). Beyond these mixed results, research on the impact of school diversity on cross-racial/ethnic friendships during adolescence compared to other developmental stages is much needed because adolescence is a critical developmental stage for forming intergroup attitudes and self-concepts (Newman & Newman, 2020; Umaña-Taylor, 2016) and notions of social justice (Karcher & Fischer, 2004). Studying how cross-racial/ethnic friendships are formed during adolescence can provide insights into their benefits for adolescent development and fostering positive intergroup interactions during this critical developmental stage to promote long-term positive individual and societal outcomes (Rastogi & Juvonen, 2019).

Most research has primarily tested the linear relation between school diversity and cross-racial/ethnic friendships, overlooking the potential non-linear effects. Policymakers and researchers have grappled with the idea of “sufficient diversity,” assuming that the desirable outcomes of diversity can be achieved when a certain level of representation balance across racial and ethnic groups is maintained (Malcom & Malcom-Piqueux, 2013). Thus, the definition of “meaningful representation” has crucial applications in school desegregation efforts (Danbold & Unzueta, 2020; Garces & Jayakumar, 2014). Research on other areas, such as the association between school racial and ethnic diversity and students’ trust, has observed a U-shaped curvilinear effect (Choi & Lee, 2021). Such observations suggest a need to explore the non-linear effects of school diversity on cross-racial/ethnic friendships to better determine the degree of diversity required in school settings to foster cross-racial/ethnic friendships. This study employs the concept of friendship diversity, referring to having a balanced and diverse group of close friends from different racial and ethnic backgrounds, rather than simply measuring the proportion of cross-racial/ethnic friends (Hooijsma & Juvonen, 2021; Munniksma et al., 2017). The latter approach could equate adolescents with all friends from a single racial and ethnic background to those with friends from diverse backgrounds, failing to capture meaningful distinctions. For example, if White Student A has two Asian and two Hispanic friends, while White Student B has four Hispanic friends, White Student A would have greater friendship diversity according to the friendship diversity concept. However, both students would have the same proportion of cross-racial/ethnic friends. Thus, assessing friendship diversity can evaluate the benefits of school diversity on cross-racial/ethnic friendships proposed by contact theory.

### The Role of Interracial Climate

Apart from understanding how a structural characteristic (i.e., school diversity) may relate to cross-racial/ethnic friendships, interracial climate—defined as practices that promote racial and ethnic group status equality and positive interracial interactions—can serve as another significant contextual predictor of friendship diversity (Green et al., 1988; Titzmann, 2014). There are different approaches to conceptualizing interracial climate, but it has generally been recognized as a multidimensional construct (Green et al., 1988; Schachner et al., 2019). Some researchers have defined this multifaceted construct as practices and norms shaped by teacher behaviors and peer interactions, respectively (Bellmore et al., 2012). Although there is limited empirical research on the impact of interracial climate on friendship diversity, a positive perception of interracial climate has been linked to reduced ethnic discrimination (Bellmore et al., 2012), better school performance and less behavioral concerns (Mattison & Aber, 2007), and more favorable perceptions of overall school climate (Green et al., 1988). A closely related construct, cultural diversity climate (i.e., equitable and inclusive school practices promoting positive intergroup contact and embracing students’ diverse cultural backgrounds as a resource) has also been shown to promote school belonging and mental health (Ahmad et al., 2018; Bardach et al., 2024) as well as positive intergroup outcomes among students (Schwarzenthal et al., 2017). Additionally, as proposed by contact theory, equal group status and positive intergroup attitudes are essential conditions for developing positive intergroup relationships in diverse settings (Pettigrew et al., 2011). Grounded in these conceptual and empirical foundations, school interracial climate would likely facilitate friendship diversity.

### The Moderating Role of Adolescents’ Immigrant Status

Guided by major developmental theories, including the integrative model for the study of developmental competencies in minority children (Coll et al., 1996) and bioecological theory (Bronfenbrenner & Morris, 2006), the developmental processes of children and youth are influenced by the intersection between individuals’ social positions (e.g., culture, socioeconomic status, race/ethnicity) and contextual factors. The current study focuses on immigration status, recognizing that cross-racial/ ethnic friendships can be a particularly valuable resource for the positive adaptation and acculturation processes of youth from immigrant families (Titzmann, 2014). Adolescents from immigrant families, however, also often face more challenges in forming cross-racial/ethnic friendships. For adolescents from immigrant families, a preference for peers sharing the same racial and ethnic background (i.e., friendship homophily) is particularly salient due to cultural and language barriers with their native-born peers (Smith et al., 2016; Titzmann & Silbereisen et al., 2009). Although friendship homophily has been found to be conducive to immigrant adolescents’ development in some respects, including more positive health outcomes and adaptation (McMillan, 2019; Titzmann et al., 2007), friendship homophily may simultaneously hinder them from developing skills needed for multicultural understanding, intergroup collaboration, and social adaptation (Titzmann, 2014).

Adolescents from immigrant and non-immigrant families may respond to school contextual factors (i.e., school diversity, interracial climate) differently, leading to varied behaviors and attitudes toward cross-racial/ethnic friendships. In the U.S., adolescents from immigrant families often possess intersecting disadvantaged positions, such as language barriers, lower socioeconomic status, cultural marginalization, limited social capital, more discriminatory experiences, and undocumented status (e.g., Metzner et al., 2022). These intersecting marginalized identities, in turn, can lead youth from immigrant families to distrust outgroup members and view them as more of a threat, contributing to a greater preference for ingroup members than their counterparts from non-immigrant families, particularly in a highly diverse school, consistent with conflict and constrict theories (e.g., McLaren, 2003). Furthermore, owing to the heightened perceptions of threat from outgroup members, having a positive interracial climate would likely be particularly important for adolescents from immigrant families, as it helps them feel safer and gain more trust that facilitates cross-racial/ethnic friendships. To better understand the roles of school diversity and interracial climate and identify strategic practices to foster cross-racial/ethnic friendships for adolescents from different backgrounds, the interaction between adolescents’ immigrant status and school contextual factors in shaping cross-racial/ethnic friendships was investigated in the current study.

## The Current Study

**Fig. 1:**
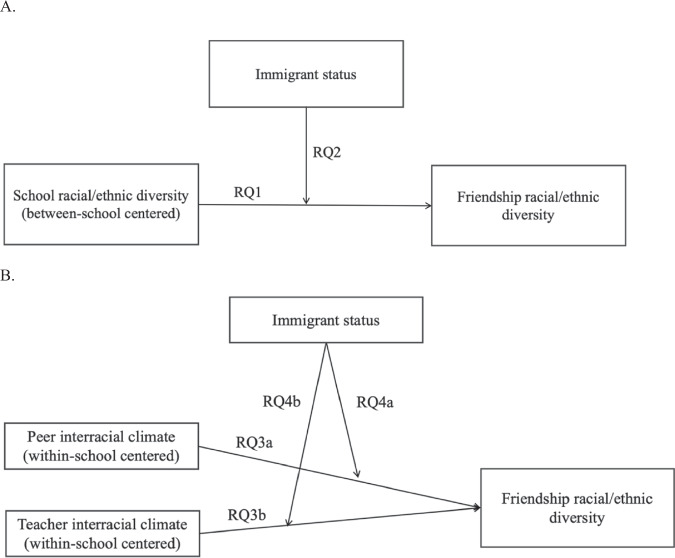
Conceptual models. **A** depicts the conceptual relation between school diversity and friendship diversity, as well as the moderating role of adolescents’ immigrant backgrounds in this association. **B** illustrates the conceptual associations between peer and teacher interracial climate and friendship diversity, as well as the moderating role of adolescents’ immigrant backgrounds. Note: only central study variables related to the research questions were depicted

Cross-racial/ethnic friendships are conducive to student development and social harmony, particularly for immigrant students, but there is limited understanding of which and how school contextual factors promote cross-racial/ethnic friendships among adolescents. Addressing this limitation, the current study examined how school diversity and interracial climate related to cross-racial/ethnic friendships with a consideration of students’ immigrant status. First, this study examined the extent to which school diversity was associated with students’ friendship diversity (Research Question 1a). The curvilinear relation of school diversity with friendship diversity was also tested, drawing upon prior findings (Research Question 1b). Given the divergence in both theory and existing evidence, a specific directional hypothesis for this relation was not offered. Second, this study assessed how immigrant status moderated the association between school diversity and students’ friendship diversity (Research Question 2). Adolescents from immigrant families were expected to have less friendship diversity in more diverse school contexts, given their potential heightened distrust towards outgroup members compared with adolescents from non-immigrant families. Third, the roles of peer interracial school climate (Research Question 3a) and teacher interracial climate (Research Question 3b) in friendship diversity were examined. It was hypothesized that a more positive perceived peer or teacher interracial climate would be related to greater friendship diversity, as proposed by contact theory. Lastly, this study examined the moderating role of immigrant status in the association between peer and teacher interracial climate and friendship diversity (Research Question 4a and Research Question 4b). Peer and teacher interracial climate were hypothesized to play a more pronounced role in the formation of cross-racial/ethnic friendships among adolescents from immigrant families, considering their intersecting marginalized identities. Figure 1 presents the conceptual models.

## Method

### Procedures and Participants

The analytical sample of the current study were drawn from a larger longitudinal study of 1,032 students from the southern United States (Project PISCES). This project investigated the effects of discrimination on adolescents’ development. Recruitment occurred in two cohorts: participants in the first cohort (46% of the sample) attended 9^th^ grade in the 2016–2017 school year, and participants in the second cohort attended 9^th^ grade in the 2017–2018 school year. Chi-square tests indicated that the two cohorts did not differ in gender identity (*χ*^2^[1] = 0.77, *p* = 0.38) or immigrant status (*χ*^2^[1] = 2.51, *p* = 0.11); however, significant differences were found for race/ethnicity (*χ*^2^[4] = 38.13, *p* < 0.001) and economic disadvantage status (*χ*^2^[1] = 27.64, *p* < 0.001). Responses from each cohort were combined in the analyses, and the effect of cohorts was controlled. Participants’ responses in Wave 1 (*n* = 751) and Wave 2 (*n* = 726) were used. Participants were in 9th grade (approximately 14 to 15 years old), a key developmental time as friendships are actively forming and play a particularly influential role in adolescent development (Güroğlu, 2022). All participants had parent consent and student assent (see Benner et al., 2024 for more information about participant recruitment), and students were given the option to complete surveys online or over the phone. Participants who had not completed the online survey after 6 weeks were mailed paper surveys to complete. Participants were compensated $25 upon completion of the survey. The Institutional Review Board of the second author’s university reviewed and approved all procedures.

Considering the research focus on the impact of school contexts on the racial and ethnic demographic compositions of students’ friendships within school, only participants who stayed in the same school from Wave 1 to Wave 2 were included, excluding 65 participants who moved school. Moreover, only schools with more than one respondent responding to the survey were included, which resulted in the exclusion of 26 schools, as these schools lacked within-school variances that affected the stability of estimating between-school variances (Hox & Maas, 2001). The analytic sample consisted of 53% females, 43% from the first cohort, and 41% adolescents from families with immigrant backgrounds. Nearly 40% of the participants qualified for the Free or Reduced-price Lunch (FRPL) program. The sample comprised participants from diverse racial and ethnic backgrounds, including Asian American (10%), Black (4%), Latino/a/x (34%), White (40%), and adolescents of other or multiple races/ethnicities (e.g., Middle Eastern, indigenous, biracial or multiethnic; 12%). The immigrant youth in the sample were born in 23 different countries across Canada, Central and South America, Africa, Europe, Asia, and Australia. Their parents originated from 55 different countries spanning the same geographic regions.

### Measures

#### School racial and ethnic diversity

School racial and ethnic diversity was assessed using the Frequentist-based Representative Diversity (FRD) Index as seen in Equation 1 (Chan & Benner, 2024).

$${Frequentist}-{based\; Representative\; Diversity}\,({FRD})\,{Index}=1-\sum \frac{\left|{{\rm{p}}}_{{\rm{i}}}-{{\rm{p}}}_{{\rm{i}}}^{{\rm{ref}}}\right|}{2}$$

p_i_ is the proportion of a given group within a target population and ${{\rm{p}}}_{{\rm{i}}}^{{\rm{ref}}}$ is the proportion of a given group in a reference population, with the reference population set by the researcher to represent maximum diversity in a given context. Here, the reference population as the ideally diverse school context with five equally distributed groups and the target population as the racial and ethnic distribution of the participants’ schools were specified (Chan & Benner, 2024). For example, in School A, where five racial/ethnic groups each comprise 20% of the student body, the FRD index would be 1, indicating the highest possible diversity. In contrast, School B, with 90% Black students and 10% White students, would have an FRD index of 0.3, reflecting lower diversity. The higher the index value is, the more similar the target school is to the ideally diverse context. In the example, School A would be more diverse than School B (see the computations of Example 1 in Appendix A). Groups included in the calculation of the index for all schools were the percentages of Black, White, Latino/a/x, Asian, and Other (i.e., American Indian, Pacific Islanders, Bi/Multiracial) students in each participating school.

#### Friendship racial and ethnic diversity

Students were asked to indicate the proportion of their close friends who were African American/Black, Latino/Hispanic, Asian American, and White on a five-point scale (0 = *None*, 1 = *A few*, 2 = *About half*, 3 = *Most*, 4 = *All*) in Wave 2. These categorical responses were then converted to corresponding percentages: None = 0%, a few = 20%, half = 50%, Most = 80%, and All = 100%. A friendship diversity score was calculated for each student using the FRD index, following a similar approach as school racial/ethnic diversity. However, the reference population for calculating friendship diversity consisted of four equally distributed racial/ethnic groups based on the possible racial and ethnic categories provided in the question. For example, if a student reported having a few Black friends, no White or Asian American friends, and most friends being Latino/Hispanic, their friendship FRD index was 0.45 (see Example 2 in Appendix A for the computations).

#### Perceived interracial climate

Two subscales from a modified version of the school interracial climate scale were used to measure students’ perceived interracial climate in interactions with peers and teachers within their school contexts (Bellmore et al., 2012; Green et al., 1988). Participants responded to these items in Wave 1. The peer interracial climate subscale was measured by four items with three reverse-coded items from the original Association subscale. An example item is “I talk to students of different ethnic groups only when I have to.” Teacher interracial climate was measured with four items that referred to teacher behaviors and school norms taken from the original Supportive Norms and Equal Status subscale. An example item is “Teachers here like students of different ethnic groups to understand each other.” The results of a confirmatory factor analysis confirmed that the scale was adequately represented as a two-factor model in the current sample, as the two-factor model fit the data well (robust CFI = 0.99, robust RMSEA = 0.01, and SRMR = 0.05). For both subscales, students responded using a five-point scale to indicate how true each statement was, ranging from 0 = *No way* to 4 = *For sure yes*. Mean scores were created to capture students’ perceived peer and teacher interracial climate, respectively. The two subscales showed adequate internal reliability as evidenced by the omega coefficients (Peer Interracial Climate: 0.68; Teacher Interracial Climate: 0.77).

#### Immigrant status

Respondents’ immigrant status was created using three items asking about participants’ and their parents’ birth country. Students were coded as 1 to denote being first- or second-generation immigrant (when they or one/both parents were born outside of the U.S.). Participants were coded as 0 if they and their parents were born in the U.S.

#### Covariates

Control variables included adolescent gender (1 = *female*, 0 = *male*), race/ethnicity (Asian, Black, Latino/a/x, other races/ethnicities with White being the reference group), economic disadvantage (1 = *qualify for FRPL*, 0 = *does not qualify for FRPL*), and cohort (1 = *cohort 1*, 0 = *cohort 2*).

### Analytic Plan

Given the nested nature of the data and research questions, a multilevel analytical framework that modeled students nested within schools was employed. All mixed effects models were examined using the lme4 package in R using restricted maximum likelihood estimations (REML; Bates et al., 2015). Model comparisons were conducted using a likelihood ratio test (LRT). Data were first screened for missingness. The Little’s test of missing at completely random (MCAR) with the study variables was conducted, and the results supported the hypothesis that data were missing at missing completely at random $\left({\chi }^{2}[1]=243,{df}=221,p=0.15\right).\,$The demographics of participants with vs without complete responses were compared using a series of chi-square analyses. The results indicated that these two groups of participants did not differ in terms of their gender identification, immigrant status, cohort, or race/ethnicity; however, significantly more non-economically disadvantaged students had completed responses $\left({\chi }^{2}=10.14,{df}=1,p=0.002,\right)$ with a small effect size (Cramer’s V = 0.10). Missing data were handled under the assumption of missing at random using listwise deletion, as the R package requires this missing data strategy. The total possible sample comprised 591 adolescents from 29 schools.

Model specification involved several steps based on theoretical and statistical considerations (Snijders & Bosker, 2011). An unconditional multilevel model was used to estimate the friendship diversity’ intraclass correlations (ICC). For the research question concerning the link between school diversity and friendship diversity (Research Question 1a and Research Question 1b), the linear and curvilinear effects of school diversity as a school-level variable on friendship diversity were estimated. To answer the second research question, a cross-level moderation between student-level immigrant students and school-level school diversity on friendship diversity was assessed by including the interaction term between school diversity and immigrant status while modeling the random slope of immigrant status. For the third research question, the direct effects of student-level peer and teacher interracial climate were examined in separate models while controlling for school diversity. These variables were centered within schools to isolate their effects from between-school variances (Research Question 3a and Research Question 3b). This study then examined whether immigrant status moderated each of these relations by adding the interaction term between immigrant status and peer or teacher interracial climate (Research Question 4a and Research Question 4b). In all models, student-level demographic variables were included and grand-mean centered. Standardized regression coefficients are reported to reflect the strength of associations.

## Results

### Preliminary Analysis

The intraclass correlation (ICCs) was 0.16 for friendship diversity, reflecting that that a significant portion of variances of friendship diversity was explained at the school level. Table 1 presents the Pearson correlation coefficients for the focal variables. Friendship diversity was positively correlated with school diversity, indicating that students in schools with higher racial/ethnic diversity reported greater friendship diversity on average. Friendship diversity was negatively correlated with students’ immigrant status, suggesting that students from immigrant families tended to have lower friendship diversity than their non-immigrant peers. School diversity was negatively correlated with both students’ immigrant status and teacher interracial climate, suggesting that schools with greater racial/ethnic diversity had a lower proportion of students from immigrant families and lower perceived school-level teacher interracial climate; however, the positive correlation between school diversity and peer interracial climate indicated that schools with higher racial/ethnic diversity reported higher positive peer interracial climate on average. There were no significant relations among either peer or teacher interracial climate and students’ immigrant status.

**Table 1:** Descriptive Statistics and Correlation Coefficients of Central Study Variables

	1	2	3	4	5
1. W2 Friendship diversity	–				
2. School diversity	0.25***	–			
3. W1 Peer interracial climate	−0.01	0.10*	–		
4. W1 Teacher interracial climate	0.02	−0.09*	0.04	–	
5. Immigrant status	−0.13**	−0.22***	−0.07	−0.04	–
*N*	603	29	591	596	631
Range	0–1	0–1	0–4	0–4	0 or 1
*M*	0.68	0.48	3.25	2.52	0.42
*SD*	0.17	0.14	0.76	0.96	0.49

Total possible student *N* = 591 for friendship diversity, interracial climate, and immigrant status. Total possible school *N* = 29 for school diversity

* *p* < 0.05. ** *p* < 0.01. *** *p* < 0.001

### Relation Between School Diversity and Friendship Diversity

**Fig. 2:**
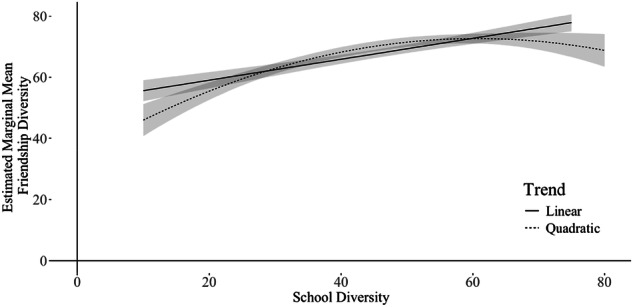
Relation Between School Diversity and Friendship Diversity

Regarding how school diversity was related to friendship diversity, the model with only a linear relation between school and friendship diversity (Research Question 1a) was compared to the model with a quadratic term added (Research Question 1b). The significant LRT result suggested that the model with the quadratic term showed a better fit (*χ*^2^(1) = 4.28, *p* = 0.04). Both the linear and quadratic effects of school diversity were significant (Linear: *β* = 0.21, *p* < 0.01; Quadratic: *β* = −0.11, *p* < 0.05) even after controlling for students’ demographic characteristics (see Table 2 Model 1a). Figure 2 visualizes the association between school diversity and friendship diversity, revealing that students attending schools with higher levels of diversity reported more racially/ethnically diverse friendship groups compared to students in schools with lower diversity; however, the positive association between school diversity and friendship diversity diminished as school diversity increased.

**Table 2:** Standardized and Unstandardized Regression Coefficients of Effects of School Diversity and Immigrant Status on Friendship Diversity

	Model 1a			Model 1b			Model 1c		
	*β*	B	SE	*β*	B	SE	*β*	B	SE
Intercept	0.14***	70.69	1.46	0.13***	70.47	1.45	0.16	70.89	1.62
School Diversity _school-level_ Linear	0.27***	0.33	0.09	0.26**	0.32	0.09	0.32***	0.40	0.10
School Diversity _school-level_ Quadratic	−0.12*	−0.01	0.00	−0.10	−0.01	0.00	−0.17*	−0.01	0.01
Female _student-level_	−0.05	−1.74	1.37	−0.05	−1.88	1.37	−0.05	−1.85	1.36
Economic Disadvantage _student-level_	0.07	2.34	1.98	0.07	2.63	1.98	0.08	2.98	1.98
Asian _student-level_	−0.00	−0.27	2.65	0.04	2.57	3.04	0.07	4.03	3.18
Black _student-level_	0.06	5.04	3.66	0.06	4.83	3.65	0.07	5.79	3.65
Latino/a/x _student-level_	0.06	2.15	1.91	0.10*	4.96	2.35	0.10	3.44	2.02
Other _student-level_	0.08	4.32	2.34	0.09*	0.05	0.02	0.10*	5.13	2.35
Cohort _student-level_	−0.05	−1.63	2.29	−0.05	−1.64	2.27	−0.03	−1.21	2.41
Immigrant _student-level_				−0.10	−3.40	1.80	−0.12	−4.00	2.02
School Diversity _school-level_ Linear x Immigrant _student-level_							−0.12*	−0.30	0.13
School Diversity _school-level_ Quadratic x Immigrant _student-level_							0.04	0.01	0.01

Model 1b vs Model 1c model comparison result: *χ*^2^ (*2*) = 6.00, *p* < 0.05

*p < 0.05. **p < 0.01. *** *p* < 0.001

### The Moderating Role of Immigrant Status in School and Friendship Diversity

**Fig. 3:**
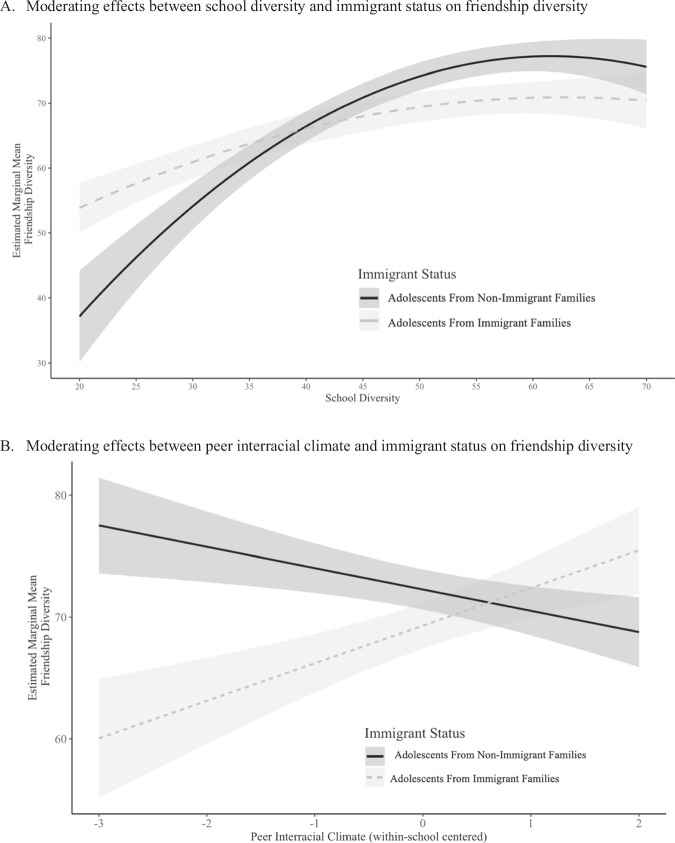
Moderating roles of immigrant status. **A** illustrates the association between school diversity and friendship diversity, presented separately for adolescents from immigrant and non-immigrant families. **B** depicts the association between peer interracial climate and friendship diversity, presented separately for immigrant and non-immigrant adolescents

Immigrant status was added to the model to examine the second research question about the moderating role of immigrant status (see Table 2). No significant differences in friendship diversity between adolescents from immigrant families versus adolescents from non-immigrant families were observed (*β* = −0.10, *p* = 0.06). For the moderating role of immigrant status (Research Question 2), the results showed that immigrant status moderated the link between school diversity and friendship diversity (*β* = −0.12, *p* = 0.02). As shown in Fig. 3A, although generally positive associations between school diversity and friendship diversity were observed for both adolescents from immigrant and non-immigrant families, the positive effect of school diversity was attenuated among students from immigrant families compared those non-immigrant families. In particular, adolescents from immigrant families reported a higher level of friendship diversity than peers from non-immigrant families in schools with low racial and ethnic diversity, whereas such pattern reversed in schools with high racial and ethnic diversity.

### School Interracial Climate and Friendship Diversity

The third research question queried the relations between students’ perceived peer and teacher interracial climates and friendship diversity and whether such relations depended on adolescents’ immigrant status (see Table 3). A direct association of either student-level peer or teacher interracial climate with friendship diversity one year later was not found, suggesting that students who perceived more positive peer or teacher interracial climate within schools did not report higher levels of friendship diversity than their peers reporting less favorable peer or teacher interracial climate in the same school. Regarding the moderating role of immigrant status, a significant interactive effect between immigrant status and within-school-centered peer interracial climate was found upon controlling school diversity and other students’ demographic characteristics (*β* = 0.10, *p* = 0.004; Fig. 3B). For the simple slopes of the moderation, adolescents from immigrant families who perceived more favorable peer interracial climate reported more friendship diversity (*β* = 2.14, *p* = 0.03), but there was not a significant association for students from non-immigrant families (*β* = −1.50, *p* = 0.08). Moderation by immigrant status was not observed for perceived teacher interracial climate.

**Table 3:** Standardized and Unstandardized Regression Coefficients of Effects of School Interracial Climate and Immigrant Status on Friendship Diversity

	Model 2a			Model 2b			Model 3a			Model 3b		
	*β*	B	SE	*β*	B	SE	*β*	B	SE	*β*	B	SE
Intercept	0.10***	70.77	1.53	0.14***	70.43	1.52	0.16***	72.04	1.65	0.14***	70.39	1.52
School Diversity _school-level_ Linear	0.28***	0.37	0.10	0.31***	0.38	0.09	0.29***	0.37	0.09	0.31**	0.39	0.10
School Diversity _school-level_ Quadratic	−0.09	−0.01	0.00	−0.10	−0.01	0.00	−0.10	−0.01	0.00	−0.10	−0.01	0.00
Female _student-level_	−0.08	−2.16	1.39	−0.06	−2.05	1.37	−0.06	−1.89	1.40	−0.06	−2.07	1.37
Economic Disadvantage _student-level_	0.08	2.82	1.99	0.07	2.58	1.98	0.08	3.05	1.99	0.07	2.56	1.98
Asian _student-level_	0.04	1.82	3.03	0.04	2.06	3.03	0.03	1.86	3.01	0.04	2.04	3.03
Black _student-level_	0.05	5.07	3.59	0.05	6.01	3.60	0.06	5.45	3.56	0.07	5.89	3.63
Latino/a/x _student-level_	0.11	3.88	2.04	0.11*	4.04	2.04	0.11	3.89	2.35	0.11	3.97	2.05
Other _student-level_	0.07	4.45	2.36	0.07*	4.86	2.35	0.08	3.88	2.37	0.09*	4.89	2.35
Cohort _student-level_	−0.07	−3.03	2.35	−0.10	−3.48	2.33	−0.09	−3.02	2.34	−0.10	−3.39	2.34
Immigrant _student-level_	−0.07	−2.90	1.80	−0.09	−2.91	1.81	−0.09	−2.96	1.18	−0.08	−2.89	1.81
Peer Interracial Climate _student-level_	0.02	−0.01	0.94				0.01	−2.10	0.94			
Peer Interracial Climate _student-level_ x Immigrant. _student-level_							0.10**	5.47	1.92			
Teacher Interracial Climate _student-level_				0.06	1.14	0.73				0.07	1.16	0.72
Teacher Interracial Climate _student-level_ x Immigrant _student-level_										0.01	0.50	1.47

Model 3a vs Model 4a model comparison result: *χ*^2^ (*1*) = 8.07, *p* < 0.01. Model 3b vs Model 4b model comparison result: *χ*^2^ (*1*) = 0.20, *p* = 0.66. Peer and teacher interracial climate were within-school centered.

*p < 0.05. **p < 0.01. ***p < 0.001

### Sensitivity Analysis

A sensitivity analysis was conducted by including all schools to assess whether excluding schools with only one participant affected the results. The findings indicated that the analytical outcomes related to the research questions remained consistent (see Supplemental Materials Tables S1–S2).

## Discussion

Cross-racial/ethnic friendships play a vital role in fostering positive intergroup interactions and social adjustment among adolescents (Kelleghan et al., 2019). However, intentional efforts are necessary to promote these friendships in schools, given adolescents’ tendencies to form friendships primarily with same-race/ethnic peers (Joyner & Kao, 2000), with this friendship homophily even more evident among adolescents from immigrant families (Smith et al., 2016). Despite this need, there remains a limited understanding of the specific school contexts that facilitate cross-racial/ethnic friendships particularly in intersection with adolescents’ immigrant status. Thus, the present study employed a multilevel framework to investigate how school contextual factors, specifically school racial and ethnic diversity and peer and teacher interracial climate, were related to the racial and ethnic composition of adolescents’ close friendship networks and how these school characteristics interacted with adolescents’ immigrant status.

First, in alignment with some prior findings, positive associations between school diversity and friendship diversity were observed (Bohman & Miklikowska, 2021; Lorenzo et al., 2024). The results empirically supported the hypothesis based on contact theory (Pettigrew et al., 2011), indicating that schools with more racially/ethnically diverse populations provide a favorable condition for making cross-racial/ethnic friendships. The results also extended the prior findings by revealing important nuances about the effects of school diversity. The positive influences of school diversity diminished after reaching a certain diversity threshold, which aligns with the concept of “sufficient diversity” (Garces & Jayakumar, 2014). More research with larger school samples with greater school diversity ranges would help pinpoint the “sufficient diversity” threshold and clarify how much diversity is needed to achieve these positive effects. These findings reinforce prior research highlighting the advantages of school integration efforts in fostering positive intergroup relationships (Lorenzo et al., 2024) and underscore the necessity for policymakers and administrators to work on diversifying school populations in the U.S.

Second, rarely has previous research considered the intersection of school contexts with students’ immigrant status on cross-racial/ethnic friendships. The current study’s findings observed that the impact of school diversity on friendship diversity also depended on students’ immigrant generational status. Contrary to the negative link between immigrant status and friendship diversity documented in some prior studies (Smith et al., 2016; Titzmann & Silbereisen, 2009), a negative relation between immigrant status and friendship diversity was not found; however, compared to adolescents from non-immigrant families, adolescents from immigrant families reported greater friendship diversity in schools with low diversity but lower friendship diversity in schools with high diversity. Moreover, the positive relation between school diversity and friendship diversity was less pronounced among adolescents from immigrant families compared with adolescents from non-immigrant families. One potential reason for these seemingly divergent findings is the differing definitions of cross-racial/ethnic friendships. Most prior research on cross-ethnic friendships and immigrant vs non-immigrant youth drew from the concept of friendship homophily (Smith et al., 2016; Titzmann, 2014) rather than friendship diversity as done in the current study. For instance, immigrant homophily in friendships was observed higher in classes with more same-racial/ethnic peers for immigrants but not native-born students (Smith et al., 2016). Thus, the current findings are not necessarily contradictory to studies focusing on homophily; rather, students from immigrant families may be more likely to make more same- and cross-racial/ethnic peers in more diverse settings where they gain more opportunities to interact with same- and cross-racial/ethnic peers.

Regarding the differential responses of adolescents from immigrant and non-immigrant families to school diversity, the friendship diversity of adolescents from immigrant and non-immigrant families may develop differently in racially and ethnically diverse settings due to varying social perceptions influenced by social position. A highly racially and ethnically diverse setting may be perceived as more threatening and uncertain for immigrant youth due to the social complexities involved, resulting in more “hunker down” behaviors compared to non-immigrant adolescents who often possess more social resources, consistent with the constrict theory (Putnam, 2007). The interactive effects of adolescents’ social position (e.g., race and ethnicity, minority status, immigrant status) and school racial/ethnic composition for cross-racial/ethnic friendship have also been documented in a few past studies (e.g., Bagci et al., 2014; Smith et al., 2016). With increasing evidence suggesting that students with different social positions respond differently to the racial and ethnic composition of their schools, more research is needed to understand the factors contributing to these varying responses.

In considering the scant empirical research on which specific aspects of school climate promote friendship diversity among adolescents, the current study further examined the roles of peer and teacher interracial climate, taking adolescents’ immigrant backgrounds into consideration. The findings showed that favorable peer interracial climate was associated with more racially and ethnically diverse friendship networks among adolescents from immigrant families but not among adolescents from non-immigrant families, partially consistent with the proposed hypothesis based on contract theory (Pettigrew et al., 2011) and the few empirically supported evidence (Ahmad et al., 2018; Bellmore et al., 2012). Conflict and constrict theories suggest that there is more distrust and perceived threat between groups in diverse contexts (Blalock, 1967; Putnam, 2007). Given the multiple disadvantaged social identities often held by immigrant adolescents (Metzner et al., 2022; Suárez-Orozco et al., 2015), it may be particularly significant for students from immigrant families to perceive their peers embracing welcoming and friendly norms to form cross-racial/ethnic friendships, as the positive peer interracial climate may reduce perceived intergroup distrust and threats and then encourage cross-racial/ethnic interactions. The mediating role of distrust and perceived threats in the relation between peer interracial climate and friendship diversity warrants further investigation to uncover the mechanisms. To help adolescents from immigrant families adapt to school environments and form diverse friendship networks, the findings imply that school administrators can work toward building an inclusive school culture that encourages peers’ openness to cross-racial/ethnic interactions and promotes respect and acceptance of various cultural backgrounds.

Interestingly, although it has been suggested that teachers play a crucial role in creating a positive school climate and promoting positive intergroup interactions (Cappella et al., 2017; Green et al., 1988), the current findings did not observe a link between teacher interracial climate and friendship diversity. It is possible that the perceived norms in peer interactions may have a more direct impact on adolescents’ friendship dynamics compared to norms fostered by teachers as shown by extensive evidence highlighting the direct impact of peer influences on friendship formation during adolescence (Laursen & Veenstra, 2021). In contrast, the influence of teachers on adolescent friendships appears to be indirect, mediated through teacher-child relationships and classroom management practices (Chen et al., 2020; Serdiouk et al., 2019). Another potential reason for the null findings relates to the study design. Adolescents’ perceptions of their teacher interracial climate from the previous academic year were used to predict their friendship diversity one year later. In U.S. high schools, students’ perceived teacher interracial climate may vary considerably from year to year because students typically interact with a new set of teachers after advancing to the next grade level. In contrast, their perceptions of interracial climate shaped by peers are likely more stable, given that they continue to interact with the same cohort of peers.

### Strengths, Limitations, and Future Directions

Using a racially and ethnically diverse adolescent sample, the present study employed a multilevel framework to examine the links among school diversity, school interracial climate, and friendship diversity, which has been proposed to promote adolescents’ intergroup attitudes as well as long-term social integration. The nature of the data enabled us to disentangle the influences exerted at the school- and student-level, offering a better understanding of how each level impacts friendship diversity. The current investigation of the interaction between school contextual factors and adolescents’ immigrant status revealed that the impact of school diversity and school practices was contingent upon adolescents’ social position. These findings render insights into reconciling the divergent perspectives concerning the role of school diversity in intergroup interactions. Additionally, school interracial climate was conceptualized as a two-dimensional construct, providing some initial empirical evidence to identify specific aspects of school climate (peer vs teacher) that facilitate friendship diversity.

Despite these strengths and contributions, some limitations should be noted. First, the study defined immigrant status based on participants’ reports of their and their parents’ birthplaces. However, this study did not examine participants’ acculturation experiences or the combined effects of immigrant status with other language and cultural factors known to affect cross-racial/ethnic friendship formation, such as perceived cultural distance and language proficiency (Schachner et al., 2015). Future research should delve deeper into how various language and cultural factors contribute to immigrant adolescents’ experiences in forming cross-racial/ethnic friendships in school. Second, perceived trust, power, and threats within school contexts may partially explain the differential effects of school diversity and interracial practices across adolescents from immigrant versus non-immigrant families, as suggested by extant theories (Pettigrew, 1998; Putnam, 2007). Future studies should investigate whether adolescents from different social positions perceived trust, power, and threats differently within the same context and whether these perceptions might mediate the impact of school contextual factors on forming cross-racial/ethnic friendships. Likewise, broader contextual factors, such as social norms and structures in different countries, could shape the influence of social positions and impact individuals’ and groups’ social interactions, potentially leading to different associations. Third, the bioecological framework proposes the dynamic interaction between different contexts in adolescent development (Bronfenbrenner & Morris, 2006); thus, examining practices in different contexts, such as cultural socialization in family and community social contexts, could reveal how these processes in various immediate settings shape adolescents’ friendship networks (Munniksma et al., 2017; Wang et al., 2020). Fourth, this study relied on self-reported perceived interracial climate, which was subject to individual experiences and subjective interpretation. With larger school samples and objective measures capturing interracial climate, future research can complement the current findings by identifying practices employed at the school or classroom level that promote cross-racial/ethnic friendships. Lastly, the current study did not inquire participants regarding their close friends’ immigrant status or other social identities. Therefore, it is unclear whether students from immigrant families tended to make friends with students also from immigrant backgrounds regardless of racial and ethnic differences. Future work utilizing qualitative methods may help further discern how social identities affect adolescents’ friendship formation.

## Conclusion

Fostering positive cross-racial/ethnic interactions in schools offers significant benefits for both individuals and society. However, school contextual factors that facilitate diverse friendships and the theoretical basis for their relation, have remained unclear. The present study employed a multilevel framework to examine the associations of school racial/ethnic diversity and peer and teacher interracial climate with friendship diversity among U.S. adolescents. The findings corroborate contact theory and reinforce prior evidence regarding the importance of school diversity in helping adolescents to form racially and ethnically diverse close friendship networks. Specifically, although school diversity generally facilitated the development of diverse friendships, there were diminishing positive effects once diversity reached a certain threshold, aligning with emerging evidence that the relation between diversity and intergroup relationships may be non-linear. Furthermore, the current study showed that the benefits of school diversity for cross-racial/ethnic friendships were less pronounced for immigrant youth than their peers from non-immigrant families. Beyond the contact opportunities provided by diversity, the findings partially substantiate contact theory by identifying the critical role of peer interracial climate as optimal condition characteristics for fostering friendship diversity among adolescents from immigrant families. The current study provides empirical evidence and directions for schools to foster cross-racial/ethnic friendships, supporting immigrant adolescents’ adaptation and promoting social harmony.

## Supplementary information

Supplementary Materials

## Appendix A

**Example 1:**

School Racial/ethnic Diversity

$${\rm{School\; A}}{\rm{\mbox{'}}}{\rm{s\; FRD\; index}}=1-\frac{\left(|0.2-0.2|\right)+\left(|0.2-0.2|\right)+\left(|0.2-0.2|\right)+\left(|0.2-0.2|\right)+\left(|0.2-0.2|\right)}{2}]=1$$

$${\rm{School\; B}}{\rm{\mbox{'}}}{\rm{s\; FRD\; index}}=1-\frac{\left[\left(\left|0.9-0.2\right|\right)+\left(\left|0.1-0.2\right|\right)+\left(\left|0-0.2\right|\right)+\left(\left|0-0.2\right|\right)+\left(\left|0-0.2\right|\right)\right]}{2}=0.3$$

**Example 2:**

Friendship Racial/ethnic Diversity

$${\rm{Friendship\; FRD\; index}}=1-[\frac{\left(|0.2-0.25|\right)+\left(|0-0.25|\right)+\left(|0-0.25|\right)+\left(|0.8-0.25|\right)}{2}]=0.45$$
