# Supplementary material for: Promoting Cross-Racial and Ethnic Friendships in Schools: Roles of School Diversity and Interracial Climate and Intersections with Immigrant Status
Source: J Youth Adolesc. 2025 Apr 3;54(7):1718–31. doi: 10.1007/s10964-025-02182-z (PMC12245967; doi:10.1007/s10964-025-02182-z)
Supplement: Supplementary file 1 — Supplementary Materials [file 10964_2025_2182_MOESM1_ESM.docx]

**Supplementary Materials**

**Promoting Cross-Racial and Ethnic Friendships in Schools: Roles of School Diversity and Interracial Climate and Intersections with Immigrant Status**

Sensitivity Analysis: Analyses including schools with only one respondent

Table S1

*Standardized and Unstandardized Regression Coefficients of Effects of School Diversity and Immigrant Status on Friendship Diversity*

|  | Model 1a | |  | Model 1b | | | Model 1c | | |
| --- | --- | --- | --- | --- | --- | --- | --- | --- | --- |
|  | $\beta$ | B | SE | $\beta$ | B | SE | $\beta$ | B | SE |
| Intercept | 0.11*** | 74.19 | 0.99 | 0.10*** | 74.03 | 0.98 | 0.14 | 70.89 | 1.62 |
| School Diversity _school-level_ Linear | 0.29*** | 0.37 | 0.08 | 0.29*** | 0.37 | 0.08 | 0.36*** | 0.40 | 0.10 |
| School Diversity _school-level_ Quadratic | -0.10* | -0.01 | 0.00 | -0.09* | -0.01 | 0.00 | -0.21*** | -0.01 | 0.01 |
| Female _student-level_ | -0.08 | -2.54 | 1.34 | -0.08* | -2.64 | 1.34 | -0.08 | -2.57 | 1.33 |
| Economic Disadvantage _student-level_ | 0.08 | 2.57 | 1.86 | 0.08 | 2.80 | 1.86 | 0.09 | 2.91 | 1.86 |
| Asian _student-level_ | -0.00 | -0.14 | 2.56 | 0.03 | 1.95 | 2.95 | 0.05 | 3.02 | 3.09 |
| Black _student-level_ | 0.05 | 4.33 | 3.39 | 0.05 | 4.20 | 3.39 | 0.07 | 5.53 | 3.38 |
| Latino/a/x _student-level_ | 0.08 | 2.95 | 1.85 | 0.11* | 3.97 | 1.98 | 0.11* | 3.90 | 1.96 |
| Other _student-level_ | 0.06 | 3.02 | 2.24 | 0.07 | 3.48 | 2.26 | 0.07 | 3.67 | 2.25 |
| Cohort _student-level_ | -0.07 | -2.34 | 1.72 | -0.07 | -2.43 | 1.71 | -0.05 | -1.57 | 1.79 |
| Immigrant _student-level_ |  |  |  | -0.07 | -2.49 | 1.77 | -0.13** | 80.05 | 24.70 |
| School Diversity _school-level_ Linear x  Immigrant _student-level_ |  |  |  |  |  |  | -0.11** | -2.78** | 0.90 |
| School Diversity _school-level_ Quadratic x Immigrant _student-level_ |  |  |  |  |  |  | 0.11 | 0.02 | 0.01 |

*Note.* Model 1b vs Model 1c model comparison result: $\chi^{2}$ (*2*) = 12.70, *p* < .01.

**p* < .05. ***p* < .01. *** *p* < .001.

Table S2

*Standardized and Unstandardized Regression Coefficients of Effects of School Interracial Climate and Immigrant Status on Friendship Diversity*

|  | Model 2a | | | Model 2b | | | Model 3a | | | Model 3b | | |
| --- | --- | --- | --- | --- | --- | --- | --- | --- | --- | --- | --- | --- |
|  | $\beta$ | B | SE | $\beta$ | B | SE | $\beta$ | B | SE | $\beta$ | B | SE |
| Intercept | 0.10*** | 74.02 | 0.97 | 0.09*** | 73.99 | 0.95 | 0.10*** | 75.08 | 1.13 | 0.09*** | 74.00 | 0.95 |
| School Diversity _school-level_ Linear | 0.28*** | 0.37 | 0.08 | 0.30*** | 0.38 | 0.08 | 0.28*** | 0.37 | 0.08 | 0.30*** | 0.38 | 0.08 |
| School Diversity _school-level_ Quadratic | -0.09* | -0.01 | 0.00 | -0.09* | -0.01 | 0.00 | -0.08* | -0.01 | 0.00 | -0.09* | -0.01 | 0.00 |
| Female _student-level_ | -0.08* | -2.71 | 1.35 | -0.08 | -2.56 | 1.34 | 0.08 | -2.57 | 1.34 | -0.08 | -2.57 | 1.34 |
| Economic Disadvantage _student-level_ | 0.08 | 2.84 | 1.86 | 0.07 | 2.54 | 1.86 | 0.04 | 2.91 | 1.85 | 0.07 | 2.57 | 1.86 |
| Asian _student-level_ | 0.04 | 2.01 | 2.95 | 0.04 | 2.02 | 2.94 | 0.05 | 2.05 | 2.94 | 0.04 | 1.97 | 2.95 |
| Black _student-level_ | 0.05 | 4.16 | 3.39 | 0.06 | 4.85 | 3.40 | 0.12 | 4.18 | 3.38 | 0.06 | 4.71 | 3.42 |
| Latino/a/x _student-level_ | 0.11* | 4.00 | 1.98 | 0.12* | 4.15 | 1.98 | 0.06* | 4.08 | 1.97 | 0.11* | 4.08 | 1.99 |
| Other _student-level_ | 0.07 | 3.48 | 2.26 | 0.08 | 3.77 | 2.26 | -0.07 | 3.12 | 2.26 | 0.08 | 3.77 | 2.26 |
| Cohort _student-level_ | -0.07 | -2.33 | 1.71 | -0.08 | -2.26 | 1.68 | -0.08 | -2.24 | 1.69 | -0.08 | -2.24 | 1.68 |
| Immigrant _student-level_ | -0.07 | -2.51 | 1.77 | -0.07 | -2.32 | 1.76 | -0.08 | -2.55 | 1.76 | -0.07 | -2.29 | 1.77 |
| Peer Interracial Climate _student-level_ | 0.02 | 0.40 | 0.91 |  |  |  | 0.02 | -1.00 | 1.14 |  |  |  |
| Peer Interracial Climate _student-level_ x Immigrant. _student-level_ |  |  |  |  |  |  | 0.08* | 3.71 | 1.84 |  |  |  |
| Teacher Interracial Climate _student-level_ |  |  |  | 0.06 | 1.25 | 0.70 |  |  |  | 0.08 | 1.27 | 0.70 |
| Teacher Interracial Climate _student-level_ x Immigrant _student-level_ |  |  |  |  |  |  |  |  |  | 0.02 | 0.49 | 1.45 |

*Note.* Model 3a vs Model 4a model comparison result: $\chi^{2}$ (*1*) = 4.03, *p* <.05. Model 3b vs Model 4b model comparison result: $\chi^{2}$ (*1*) = 0.12, *p* = 0.73. Peer and teacher interracial climate were within-school centered.

**p* < .05. ***p* < .01. ****p* < .001.
